# EDC-YOLO-World-DB: A Model for Dairy Cow ROI Detection and Temperature Extraction Under Complex Conditions

**DOI:** 10.3390/ani15233361

**Published:** 2025-11-21

**Authors:** Hang Song, Zhongwei Kang, Hang Xue, Jun Hu, Tomas Norton

**Affiliations:** 1 College of Engineering, Heilongjiang Bayi Agricultural University, Daqing 163319, China; 2 Daqing Normal University, Daqing 163712, China; 3 M3-BIORES Research Group, Division of Animal and Human Health Engineering, Faculty of Bioscience Engineering, Katholieke Universiteit Leuven (KU Leuven), Kasteelpark Arenberg 30, 3001 Leuven, Belgium

**Keywords:** dairy cow, complex condition, illumination, temperature extraction, infrared thermography

## Abstract

Body temperature serves as a crucial indicator of dairy cow health. Traditional rectal temperature (RT) measurement often induces stress responses in animals. Body temperature detection based on infrared thermography (IRT) offers non-invasive and timely advantages, contributing to welfare-oriented farming practices. However, automated detection and temperature extraction from critical cow regions are susceptible to complex illumination, black-and-white fur texture interference, and region of interest (ROI) deformation, resulting in low detection accuracy and poor robustness. To address this, this paper proposes the EDC-YOLO-World-DB framework to enhance detection and temperature extraction performance under complex illumination conditions. First, URetinex-Net and CLAHE methods are employed to enhance low light and overexposed images, respectively, improving structural information and boundary contour clarity. Subsequently, spatial relationship constraints between LU and AA are established using five-class text priors—lower udder (LU), around the anus (AA), rear udder, hind legs, and hind quarters—to strengthen the spatial localisation capability of the model for ROIs. Subsequently, a Dual Bidirectional Feature Pyramid Network architecture incorporating EfficientDynamicConv was introduced at the neck of the model to achieve dynamic weight allocation across modalities, levels, and scales. Task Alignment Metric, Gaussian soft-constrained centroid sampling, and combined *IoU* (*C_IoU_* + *G_IoU_*) loss were introduced to enhance sample matching quality and regression stability. Results demonstrate detection confidence improvements by 0.08 and 0.02 in low light and overexposed conditions, respectively; compared to two-text input, five-text input increases *P*, *R*, and *mAP50* by 3.61%, 3.81%, and 1.67%, respectively; Comprehensive improvements yielded *P* = 88.65%, *R* = 85.77%, and *mAP50* = 89.33%—further surpassing the baseline by 2.79%, 3.01%, and 1.92%, respectively. Temperature extraction experiments demonstrated significantly reduced errors for $T_{Max}$, $T_{Min}$, and $T_{avg}$. Specifically, for the mean error of LU, $T_{Max}$, $T_{Min}$, and $T_{avg}$ were reduced by 66.6%, 33.5%, and 4.27%, respectively; for AA, $T_{Max}$, $T_{Min}$, and $T_{avg}$ were reduced by 66.6%, 25.4%, and 11.3%, respectively. This study achieves robust detection of LU and AA alongside precise temperature extraction under complex lighting and deformation conditions, providing a viable solution for non-contact, low-interference dairy cow health monitoring.

## Simple Summary

This paper proposes a dairy cow region of interest (ROI) detection and temperature extraction method that integrates deep learning with infrared thermography (IRT), improving model accuracy and robustness under complex conditions. The method overcomes challenges from complex illumination, interference caused by black-and-white fur texture, and performance degradation due to ROI deformation. Experiments demonstrate higher accuracy and stability in ROI detection and temperature extraction, enabling non-invasive, low-interference body temperature acquisition and health monitoring of dairy cows.

## 1. Introduction

As dairy farming develops towards intelligence, the level of automation in farming systems has continuously improved, with the demand for real-time monitoring of dairy cow health advancing significantly [1]. Rectal temperature (RT) is a crucial physiological indicator for evaluating the health of dairy cows. Changes in RT can directly reflect health problems such as fever, stress, infection, inflammation, and metabolic disorders. In disease states, the RT of dairy cows usually rises significantly. For example, during experimental mastitis infection, RT begins to increase approximately 4 h after infection, reaches a peak at around 13 h, and then gradually declines [2]. In addition, body surface temperature detected by infrared thermography (IRT) shows a similar upward trend for RT [3]. Therefore, the timely and accurate acquisition of RT information is crucial for reducing disease transmission, improving production performance, and ensuring the wellbeing of dairy cows [4].

Dairy cows generate substantial metabolic heat because of high feed digestion demands and milk synthesis, whereas their sweating capacity is relatively limited; consequently, thermoregulation relies heavily on sensible heat loss through conduction, convection, and radiation, as well as evaporative heat loss from the skin and respiratory tract [5,6]. When the ambient temperature-humidity index exceeds the effective heat-dissipation threshold, peripheral vasodilation increases blood flow to superficial tissues and leads to marked rises in body surface temperature, particularly in anatomical “thermal windows” where the skin is thin and richly vascularised [7,8]. Regions around the udder and hindquarters have been reported to exhibit pronounced temperature changes under heat stress and disease conditions [9]. These physiological characteristics indicate that temperature dynamics at such skin sites are strongly associated with systemic heat load and rectal temperature, supporting the use of local surface temperature as a non-invasive indicator of health status in dairy cows.

Traditional RT measurement methods typically involve inserting a mercury rectal thermometer into the rectum of dairy cows via the anus to measure the body temperature [10]. Despite its widespread use in clinical practice, this method is time consuming and labour intensive, and frequently invasive human–animal contact can easily trigger stress responses in animals, which is detrimental to ensuring animal welfare standards in livestock farming [11,12]. Research has revealed a high correlation between the body surface temperature and rectal temperature in dairy cows [13]. Furthermore, multi-site body temperature modelling can partially replace the traditional rectal temperature measurement method [14]. IRT is a non-contact temperature measurement technique that enables body temperature to be measured without triggering any stress responses in dairy cows [15]. Currently, IRT has been employed in studies on temperature or disease detection in various animals, including sheep, dogs, pigs, and dairy cows [16,17,18,19].

Deep learning and image processing technologies have become indispensable tools in artificial visual recognition and detection. Mainstream deep learning detection techniques include MobileNet [20], SSD [21], fast-rcnn [22], and You Only Look Once (YOLO) [23]. Among these, the YOLO series of detection models offer advantages such as lightweight design and high accuracy. Zhang et al. [24] improved the YOLO detection framework to automatically detect the temperature of sheep eyes in IRT images using SE-SC-YOLOv7 and realised non-contact physiological detection. The improved mAP of 0.5:0.95 corresponded to an increase by 13.82% compared to the previous version, and the mean absolute error (MAE) of the maximum eye temperature decreased by 16.24%. Wang et al. [25] improved YOLOv4 using GhostNet [26], Depthwise separable Convolution (DepC) [27], and GCNet [28], and combined the improved model with IRT to develop a system for automatically detecting the temperature of cows’ eyes, achieving an mAP of 96.88% and an F1 score of 91.75%.

Despite considerable progress in temperature extraction and region of interest (ROI) detection, issues such as ROI detection, temperature extraction, and RT prediction under extreme lighting conditions remain in the preliminary exploration stage [29,30]. These problems are critical to achieving all-weather RT prediction. The main challenges are described in detail below.

Firstly, RGB images are highly susceptible to variations in lighting conditions, which can lead to blurred ROI boundaries and weakened texture. This results in feature instability, increased rates of false positives and false negatives, and diminished cross-scenario generalisation capabilities. Secondly, traditional modelling treats each ROI as an independent category, causing the model to only learn superficial information, such as shape, colour, and texture, while rendering it incapable of expressing the potential semantic relationships between them [31]. For cow skin regions with significant and irregular colour changes, this ROI detection method is easily affected by colour variations, thereby reducing detection accuracy [32]. In fact, different ROIs have clear spatial and structural associations within the body structure of a cow. These structural connections help improve the detection performance of the model for multiple ROIs and ability to understand [33], particularly in cases of blurred boundaries or structural overlaps, where robustness and discrimination capabilities are enhanced.

**Figure 1:**
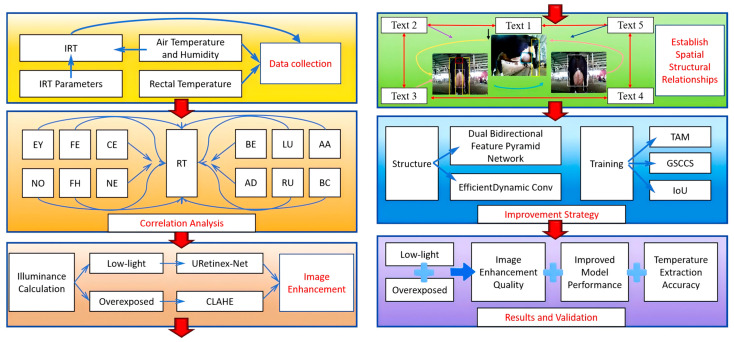
Overall architecture of this study.

The main contributions of this study are as follows, text in red indicates the main processing stages and analysis modules in the workflow. (Figure 1):

1. A physical modelling-based dual-path image enhancement method is employed to improve the structural information and boundary contours of low-light and over-exposed images.
2. Within the model, textual priors are employed to establish spatial structural relationships between irrelevant ROIs. Text-guided feature alignment and relational modelling then constrain cross-regional semantic consistency, thereby enhancing the robustness of detection and localisation.
3. An Efficient Dynamic Convolution structure is introduced to improve the network and correlation difference between different ROI image information and semantic information. Concurrently, a Dual Bidirectional Feature Pyramid Network (DB) structure is adopted to achieve cross-level feature fusion of dual-path information flow, enabling the model to better recognise fine-grained features.
4. Considering the deformability central symmetry of the ROI structure, this study optimises the training strategy by employing a task alignment metric (TAM) to enhance semantic feature learning, introducing Gaussian soft-constrained centre sampling to improve adaptability to deformable regions, and designing a hybrid *IoU* loss (*C_IoU_* + *G_IoU_*) to stabilise bounding box regression under blurred boundaries and target shifts.

## 2. Materials and Analysis

### 2.1. Data Collection

**Figure 2:**
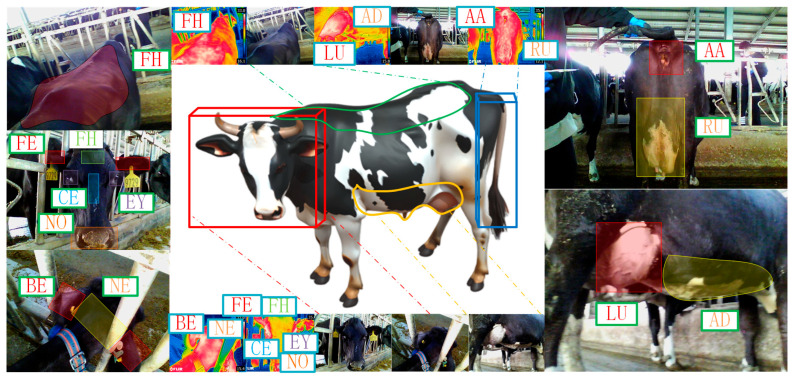
Characteristic areas on the body surface of dairy cow. The abbreviations for the various body regions of the cow are as follows: cow’s eyes (EY), nose (NO), front ears (FE), forehead (FH), centre of eyebrows (CE), neck (NE), back ears (BE), abdomen (AD), lower udder (LU), rear udder (RU), and around the anus (AA), RT.

The data collection locations and time periods for this study were as follows: 8511 dairy farm in Mishan City, Heilongjiang Province, China, from October 2023 to October 2024; and ELVO dairy barn in Belgium, in April 2025. The data collection subjects included 180 Holstein lactating dairy cows. The data collection equipment included an FLIR-E6 IR imaging camera (Teledyne FLIR LLC, Wilsonville, OR, USA) (Figure 2), a veterinary mercury thermometer (EWHA Co., Ltd., Liaocheng, Shandong, China), and a handheld temperature and humidity meter (Shandong Renke Measurement & Control Technology Co., Ltd., Jinan, Shandong, China). The cows were housed in a lactating cow barn. During each data collection session, the cows were secured to the neck rail to restrict their movement and minimise interference from other cows while feeding.

The FLIR-E6 infrared imaging camera was used to collect surface temperature data from the dairy cows. Temperature and humidity data collected by handheld thermohygrometers are used to calibrate the parameters of the infrared imaging camera. The veterinary mercury thermometer was used to collect RT from dairy cows, with a measurement range of 35–43 °C and an accuracy of ±0.1 °C. The parameter settings for the IR thermal imager are detailed in Table 1.

The data collection procedure followed a standardised protocol:

1. Each cow was restrained at the feed bunk using a neck rail;
2. A handheld thermo-hygrometer was placed at a fixed position close to the cow and kept stationary;
3. A veterinary mercury thermometer was inserted approximately 5 cm into the rectum to measure rectal temperature;
4. After about 5 min, when the readings of the thermo-hygrometer and mercury thermometer had stabilised, the air temperature and humidity parameters of the infrared thermal imager were adjusted according to the current ambient conditions, and full-body thermal images were acquired from the rear and lateral views at a distance of approximately 1.5 m from the cow;
5. After IRT image acquisition, the corresponding rectal temperature value was recorded;
6. If the cow moved or the image was blurred, the measurements were repeated until a clear and complete thermal image was obtained.

### 2.2. Dataset Structure

This study collected a total of 3854 RGB images for training the ROI detection model. The quantities of low-light, normal-light, and overexposed images are shown in Table 2. The dataset was partitioned into training, validation, and test sets in a 6:2:2 ratio.

### 2.3. Data Analysis and ROI Selection

**Figure 3:**
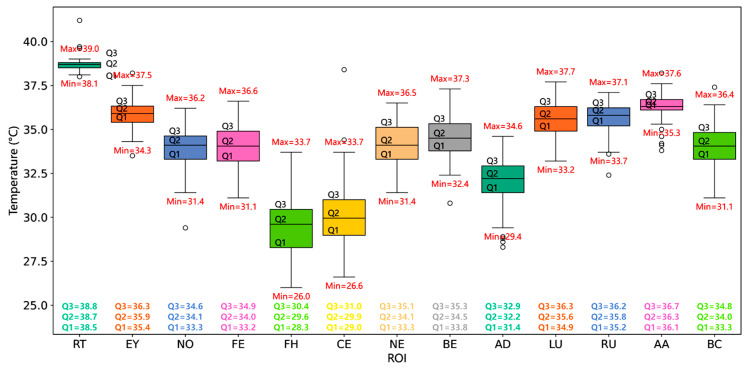
Dairy cow body surface temperature distribution map. In the box plot, Q1, Q2, and Q3 represent the first, second, and third quartiles, respectively. Different colours are used to distinguish the ROIs, and the open circles indicate outliers.

The feature regions were labelled via manual annotation, and then the body temperature was extracted within those areas. The surface temperature of dairy cows is higher than that of the environment, ground, walls, and feeding equipment. Therefore, utilising thermal imaging cameras to extract the maximum temperature within each ROI as the temperature characteristic of the cow’s body surface can avoid unnecessary errors (Figure 3).

To directly illustrate the relationship between core body temperature and peripheral surface temperatures, ten representative cows were selected, and their RT, LU surface temperature, and AA surface temperature are listed in Table 3 for comparison.

**Figure 4:**
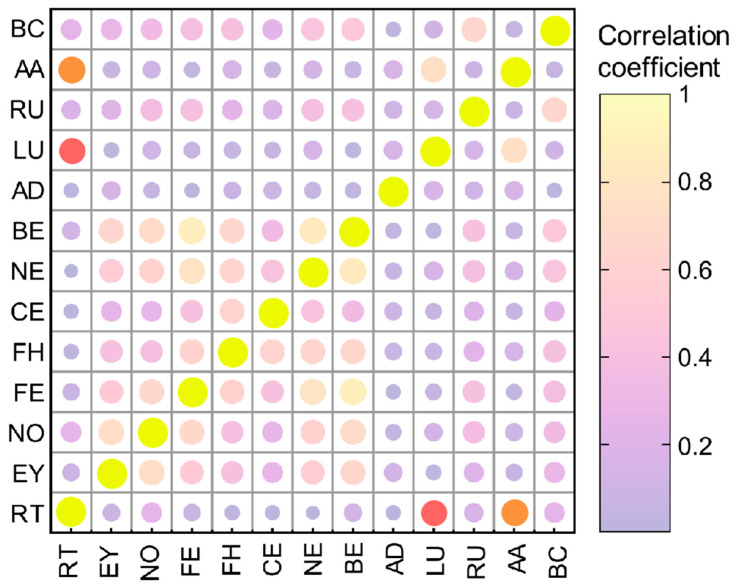
Spearman correlation heat map of body temperature in different parts of dairy cows. The size of the circle indicates the strength of the correlation.

The statistical relationships between RT and other body surface temperature measurement points in dairy cows were investigated. This was achieved using the Spearman rank correlation coefficient [34] to analyse the collected data for correlation analysis and plotting a confusion matrix of correlations between various body surface temperatures (Figure 4).

The results show that LU and AA temperatures exhibit the highest correlations with RT. Therefore, LU and AA temperatures were selected as the ROIs for surface temperature measurements in dairy cows. Therefore, LU and AA were employed as ROIs under different illuminance conditions to construct the detection model.

## 3. Methodology

### 3.1. Dual-Path Image Enhancement Method

#### 3.1.1. Light Intensity Calculation

**Figure 5:**
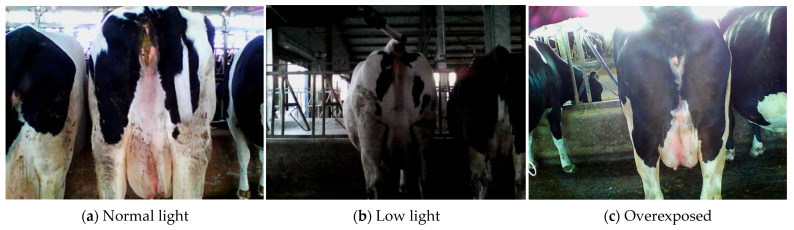
RGB images of dairy cows under varying light intensities.

When taking pictures of dairy cows in a cowshed, there is often low light and overexposure (Figure 5). We employed a method based on grey-scale statistical features to automatically determine whether an image belongs to a low-light environment or is overexposed. Equations (1)–(3) describe the grey-scale conversion method, average brightness calculation, and exposure degree criteria, respectively.

(1) $$I_{gray}=0.299\times R+0.587\times G+0.114\times B$$

(2) $$\mu =\frac{1}{N}\sum_{i=1}^{N}I_{gray}(i)$$

(3) $$Illumination\left(I\right)=f\left(x\right)=\left\{\begin{array}{ccc} \mathrm{Low-light}, & & \mu <T_{low}\\ Overexposed, & & \mu \geq T_{high}\\ \mathrm{Normal-light}, & & others \end{array}\right.$$

Firstly, the input RGB image was converted into a greyscale image (Equation (1)). Subsequently, the overall illumination level of the image was estimated by calculating the average value of all pixels in the greyscale image (Equation (2)). Finally, the illumination level of the image was classified based on a threshold (Equation (3)). In the equation, $I_{gray}$ represents the greyscale image, *μ* represents the average brightness, *N* indicates the total number of pixels in the image, and $I_{gray}(i)$ represents the greyscale value of the *i*^th^ pixel. In this study, $T_{low}$ and $T_{high}$ were set as 60 and 150, respectively.

#### 3.1.2. Low-Light Cow Image Enhancement Based on URetinex-Net

URetinex-Net is a deep unfolding network based on the Retinex theory [35]. It features excellent theoretical interpretability, strong detail preservation capabilities, and high adaptability to lighting conditions. In barn environments, wall obstructions or low-light conditions at night can result in insufficient brightness, blurred details and noise interference when images of cows are captured, thereby affecting the accuracy of subsequent image-based recognition or health assessment tasks. Therefore, this study introduces the URetinex-Net model to enhance low-light images and improve the overall image quality. In the proposed method, the image enhancement problem is modelled as the decomposition of a reflection map R and an illumination map L (Equation (4)):

(4) I(x)=R(x)·L(x)

where I(x) represents the original low-light image; R(x) represents the reflectance component that describes the intrinsic colour and texture of the body surface of the cow; L(x) represents the illumination component, which reflects the local light intensity received by each pixel in the scene.

**Figure 6:**
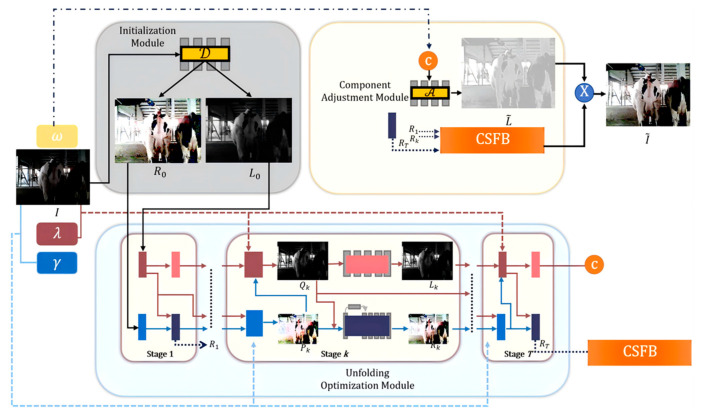
Low-light image enhancement process based on URetinex-Net.

URetinex-Net comprises an initialisation module, unfolding optimisation module, and a lighting adjustment module (Figure 6).

The initialisation module estimates the initial reflectance map ${R\;}_{0}$ and illumination component ${L\;}_{0}$ (Equation (5)) from the input image I.

(5) $$D(I)=\arg\underset{R_{0},L_{0}}{\min}\left({\left\|I-R_{0}\cdot L_{0}\right\|}_{1}+\mu \|L_{0}-\underset{\in \left\{R,G,B\right\}}{\max}I^{\left(c\right)}{\|}_{F}^{2}\right)$$

Here, D(I) represents the initialisation module, ${\left\|\cdot \right\|}_{1}$ denotes $l_{1}$ norm, μ is a hyperparameter, and $c\in \left\{R,G,B\right\}$ represents the RGB channels. Among these, the first term refers to the reconstruction loss, and the second term preserves the overall structure of the input image in the initialised lighting component $L_{0}$, where μ is a hyperparameter.

The initialised $L_{0}$ and $R_{0}$ are fed into the unfolding optimisation module. Four variables are updated at each stage, namely $P_{K}$, $Q_{K}$, $R_{K}$, and $L_{K}$, before being iterated through T stages to alternately optimise the reflection map and lighting map (Equation (6)):

(6) $$\varepsilon \left(P,Q,R,L\right)={\left\|I-P\cdot R\right\|}_{F}^{2}+\alpha \phi \left(R\right)+\beta \psi \left(L\right)+\gamma {\left\|P-Q\right\|}_{F}^{2}+\lambda {\left\|Q-L\right\|}_{F}^{2}$$

where ε(P,Q,R,L) represents the total energy function, and ϕ(·) and ψ(·) represent the regularisation terms of the reflectance map and illumination map, respectively. γ and λ are penalty parameters.

The illumination adjustment module performs non-linear enhancement on the illumination map $L_{T}$ output from the previous module, outputs the adjusted illumination map $\tilde{L}$ through a specified brightness coefficient ω, and combines it with the reflection map R to generate the final enhanced image (Equations (7) and (8)).

(7) $$\tilde{L}=A(L,\omega ;\theta_{A})$$

(8) $$\tilde{L}=R\cdot \tilde{L}$$

In the formula, A(·) represents the learnable convolutional network, $\theta_{A}$ refers to its parameter; ω represents a scalar or a mapping map with the same dimension as L, used to control the enhancement degree. In this study, ω=0.5 was taken as the enhancement parameter for low-light cow images.

#### 3.1.3. CLAHE-Based Overexposed Cow Image Enhancement

**Figure 7:**
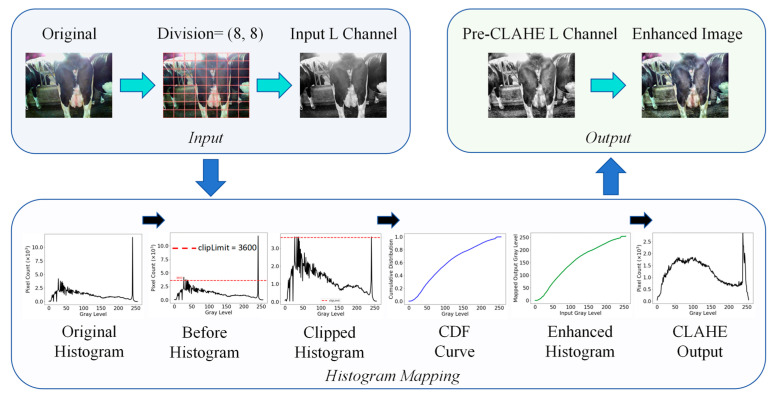
Process flow of CLAHE. The red dashed lines in the histograms indicate the clipping threshold used in CLAHE (clipLimit = 3.0, corresponding to a maximum bin frequency of 3600 pixels).

Contrast-limited adaptive histogram equalisation (CLAHE) was employed to enhance the brightness channel (L channel) (Figure 7) and suppress overexposed areas caused by local strong light in the cow image [36]. The entire process flow can be divided into three stages, as detailed below.

First, the brightness channel (L channel) is extracted from the original image in the Lab colour space as the input basis for CLAHE. Subsequently, the image is divided into 8 × 8 grid cells to separately perform histogram equalisation in each local region. In the second stage, the brightness channel histogram is calculated for each region, and pixel frequency clipping and uniform redistribution are performed based on the clipLimit (set to 3.0 in this study). Subsequently, the cumulative distribution function is constructed and normalised to 0–255 to serve as the mapping function for pixel greyscale, ultimately generating the contrast enhancement lookup table required for CLAHE. CLAHE introduces a bilinear interpolation strategy in the output stage to ensure smooth transitions at the boundaries of sub-blocks. The pixel values of a pixel point are weighted and fused based on the balanced pixel values of the four adjacent sub-blocks it belongs to. The calculation expression is as follows:

(9) $$I\left(x,y\right)=\sum_{i\epsilon (a,b,c,d)}\omega_{i}\cdot I_{i}(x,y)$$

where $I_{i}(x,y)$ represents the final greyscale value of pixel (x,y) in the output image, $I_{i}(x,y)\;$ represents the balanced result of this pixel in the *i*^th^ sub-region, and $\omega_{i}$ denotes the bilinear interpolation weight calculated based on the distance between the pixel and the centre of the sub-region. Finally, the enhanced brightness channel is recombined with the chrominance channels (a and b) of the original image and converted back to the RGB colour space to obtain the final CLAHE-enhanced image. In this study, CLAHE was used to suppress overexposed areas caused by strong light in cow images. The histogram frequency was set to 3600 pixels, with the cropping size as tileGridSize = (8, 8).

### 3.2. Improvements to YOLO-World Based on Reinforced Spatial and Structural Relationships

#### 3.2.1. The Spatial Structural Relationship Between Text and ROI

In traditional object detection, LU and AA are commonly encoded using one-hot encoding (e.g., [0,1] and [1,0]) as labels, which the model treats as completely unrelated categories. Traditional detection models rely on shape, colour, and texture features for recognition. However, the random distribution of black-and-white patterns on the body of a cow can easily confuse the model, particularly under poor lighting conditions, where strong colour contrasts exacerbate misclassification. Therefore, incorporating semantic text provides additional spatial structural information to the image, enabling the model to establish spatial relationships between ROIs and thereby improve localisation accuracy [37].

**Figure 8:**
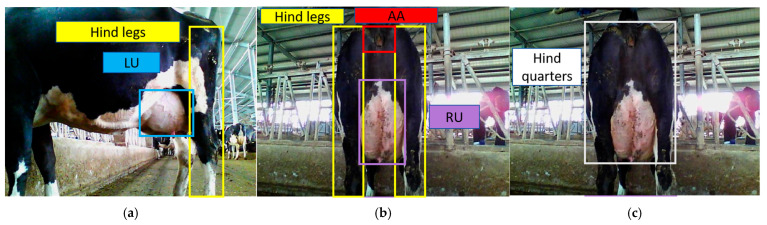
Spatial locations of each text category in the image. (**a**) side view showing the spatial relationship between the LU and the hind legs; (**b**) rear view showing the relative positions of the AA, RU, and hind legs within the hindquarters; (**c**) hindquarters region used as the coarse spatial frame for defining the text categories.

Specifically, the LU, AA, RU, hind legs, and hind quarters were defined as five text categories, each accompanied by a corresponding textual description. The ROIs in this task are LU and AA, which, if encoded as independent detection classes, do not exhibit any explicit spatial relationship. To establish a spatial structure between them, the hind quarters, hind legs, and RU were used as intermediate linking regions. From the rear view, the hind quarters provide a coarse spatial frame: within this region, the hind legs are distributed on both sides, while RU and AA lie between them, with AA located above RU. From the side view, LU is consistently adjacent to one of the hind legs. Thus, a continuous anatomical chain is formed—LU → hind legs → hind quarters (including RU and AA)—which establishes a spatial connection between LU and AA and enables the model to learn their relative positions rather than treating each ROI as an isolated category (Figure 8, Table 4).

#### 3.2.2. EDC Module

**Figure 9:**
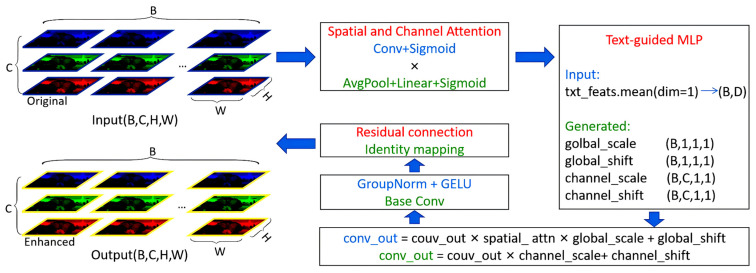
Network structure of EDC module.

In this study, the association between semantic labels and image information was enhanced using EDC module to generate dynamic convolution parameters associated with text semantics [38]. This module mainly contains three parts: a space-channel attention mechanism, a text-guided dynamic parameter generator, and a residual convolution structure (Figure 9).

The input feature map is Equation (10):

(10) $$X\in R^{B\times C\times H\times W}$$

where *B* represents the bitch size, *C* represents the number of channels, and *H* and *W* represents height and width of the feature map, respectively.

First, the module introduces a combination mechanism of spatial attention and channel attention to improve how the network perceives input feature maps. The module achieves the most weighted distribution of image space and semantic channel dimensions via channel-wise average pooling and Sigmoid function to calculate weights. The attention-refined feature map is computed as Equation (11):

(11) $$X^{\prime }=X⊗A_{S}⊗A_{C}$$

where ⨂ represents element-wise multiplication, $A_{S}$ emphasises important spatial locations, and $A_{C}$ emphasises important channels.

Text features of each ROI are encoded as Equation (12):

(12) $$\mathrm{txt\_feats}\in R^{B\times L\times D}$$

where L is the number of tokens and *D* is the test feature dimension. An average operation along the token dimension produces a compact text vector as Equation (13):

(13) $$t\in R^{B\times D}$$

This is input to a text-guided MLP [39]. This network outputs four modulation parameters: the global scale $g_{s}$, the global shift $g_{b}$, the channel-wise scale $c_{s}$, and the channel-wise shift $c_{b}$. These quantities are used to adjust the attention-refined feature map as Equation (14):

(14) $$\hat{X}=X^{\prime }⊗g_{s}⊗c_{s}+g_{b}+c_{b}$$

where $\hat{X}$ denotes the dynamically modulated feature map that is controlled by the text information.

The modulated feature maps are then normalised and passed through a GELU activation, and a residual connection is added so that the original structural information of the backbone features is preserved.

The text database is organised at the level of anatomical regions. Each entry corresponds to one of the five text categories, namely LU, AA, RU, hind legs, and hind quarters. For every entry, the database stores the category label, a short textual description, and the index of the associated image or ROI. These region-level text records are encoded into text features and are used by the text-guided multilayer perceptron to generate the dynamic modulation parameters in EDC module.

#### 3.2.3. Neck Structure Based on Dual Bidirectional Feature Pyramid Network (BiFPN)

To enhance the cross-scale fusion of multi-modal features, this study introduces the DB [40] structure into the YOLO-World framework and proposes an enhanced bidirectional multi-modal fusion module, namely Enhanced-YOLO-World-DualBiFPN.

First, a bidirectional fusion path is designed based on BiFPN. DB introduces a more flexible bidirectional connection mechanism, which includes top-down and bottom-up information flow paths. Each layer of feature maps can obtain information from both the top and bottom, combining contextual semantics and local details to achieve more comprehensive feature reuse and completion. Moreover, the learnable fusion weight mechanism of DB can transform the weight allocation method from weighted averaging to dynamic learning, enabling the model to better distinguish fine-grained structures of black-and-white contours on cow skin. Finally, EDC module is embedded to achieve semantically guided dynamic adaptation.

**Figure 10:**
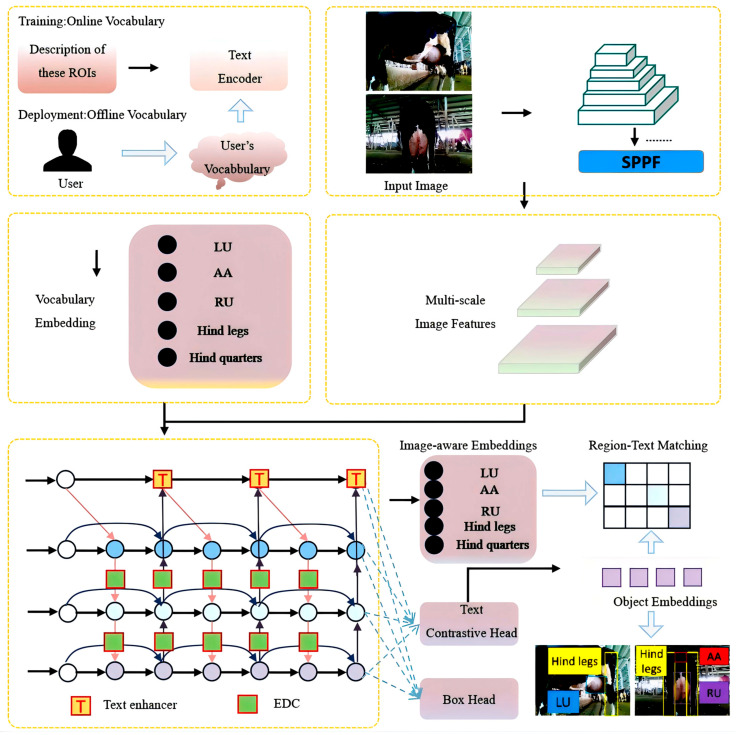
Network structure of the EDC-YOLO-World-DB model.

EDC is embedded as a dynamic adapter module at the fusion node of DB to further improve the fusion effect between images and text. This module dynamically generates parameters (global and channel-wise scale/shift) for modulating visual features based on the hierarchical level of image features and the current text prompt content, thereby achieving cross-scale semantic self-adaptive enhancement. The overall architecture of the improved model EDC-YOLO-World-DB is illustrated in Figure 10.

### 3.3. Improvements to Training Strategies

Cow images are severely affected by lighting conditions, and the ROI regions exhibit distinct structural characteristics. The LU region undergoes significant deformation owing to pose variations. The AA exhibits central symmetry. This study optimises the training strategy to make the model focus more on the above problems during training. The optimisation method uses three key mechanisms, TAM, Gaussian soft-constrained centre sampling, and mixed *IoU* metrics, to effectively improve the robustness and accuracy of the model under complex lighting and structural changes.

#### 3.3.1. Task Alignment Metric

Traditional YOLO detectors use *IoU*-based heuristic positive sample matching strategies that have difficulty coordinating the consistency of classification and regression tasks, easily resulting in insufficient positive sample quality and unstable gradients. This study introduces TAM to improve the sample allocation method of YOLO-World [41]. By integrating the classification confidence and localisation accuracy of anchors, a joint scoring function (Equation (15)) is obtained to enhance the coordination between classification and localisation:

(15) $$A_{ij}=P_{cls}^{\alpha }\cdot {IoU}_{ij}^{\beta }$$

where $P_{cls}$ denotes the classification score, and ${IoU}_{ij}$ indicates the overlap degree between the candidate box and the target. This metric prioritises anchors that perform well as positive samples in both classification and localisation, thereby improving the detection accuracy of complex target boundaries (such as pattern junctions) and balancing semantic and geometric matching by providing a more tolerant alignment strategy for easily deformable targets in the LU region.

#### 3.3.2. Gaussian Soft-Constrained Centre Sampling

Changes in cow posture significantly affect the LU region. Traditional fixed-threshold centre sampling strategies struggle to accurately capture its central region. This study addresses this by introducing a Gaussian soft-constraint mechanism to achieve dynamic weight allocation for candidate boxes [42]. This study introduces a Gaussian soft-constrained centre sampling mechanism that constructs dynamic spatial weights by introducing normalised centre distance $d_{ij}$ and a Gaussian function to increase the probability of anchor point matching near the ground truth centre:

(16) $$\omega_{ij}=exp(-\frac{d_{ij}}{2\sigma^{2}})$$

where σ is the hyperparameter that controls the sampling range. Unlike traditional hard threshold-centred sampling, this mechanism enables candidate boxes that are not precisely centred to compete for positive samples, which is beneficial for training stability when the LU area is deformed. Concurrently, the Gaussian kernel enhances the matching probability of anchor points near the centre structure of the AA, improving detection focus.

#### 3.3.3. Mixed *IoU* Loss Function

Traditional *IoU* metrics each have their own advantages and disadvantages: *G_IoU_* compensates for non-overlapping areas but has poor convergence, while *C_IoU_* introduces centre distance and aspect ratio constraints, which optimise in a clear direction but have poor robustness to occlusion and targets outside the boundary [43]. Under poor lighting conditions, object boundaries in images become blurred and scale distortion is severe. To improve the robustness of the model under these conditions, this paper introduces a weighted fusion of *C_IoU_* and *G_IoU_* as the bounding box regression loss function:

(17) $$L_{IoU}=\lambda \cdot C_{IoU}+(1-\lambda )\cdot G_{IoU}$$

where $C_{IoU}$ comprehensively considers the centre distance between the predicted box and the ground truth box, overlapping area, and aspect ratio. $G_{IoU}$ is used to penalise predicted bounding boxes that do not overlap but have a large surrounding area, and λ is the weighted coefficient between $C_{IoU}$ and $G_{IoU}$. This strategy balances the geometric shape of the target (i.e., $C_{IoU}$) with the boundary guidance capability of non-overlapping areas (i.e., $G_{IoU}$), enhancing the robustness of the model in scenarios with blurred boundaries and target shifts. In particular, it improves the localisation of AA in overexposed images and udder regions in low-light conditions.

### 3.4. Evaluation Criteria

This study uses *P*, *R*, mean average precision (*mAP*), and confidence score to evaluate the performance of the detection model:

(18) $$P=\frac{TP}{TP+FP}\times 100\%$$

(19) $$R=\frac{TP}{TP+FN}\times 100\%$$

(20) $$AP=\int_{0}^{1}P(r)dr$$

(21) $$mAP=\frac{1}{\left|C\right|}\sum_{c\epsilon C}AP_{\left(c\right)}$$

(22) $${Conf}_{i}=max\left\{S_{i,1},\;S_{i,1},\;\ldots ,\;S_{i,k}\right\}$$

where TP indicates the number of correct detections of ROIs on the body surface of the cow, FP indicates the number of false detections, and FN indicates the number of ROIs detected as other categories. AP indicates the accuracy of a category within the entire R range, c denotes the target category, mAP indicates the average of all categories of APs, and $\left|C\right|$ indicates the total number of detected target categories. *K* denotes the number of classes and $S_{i,k}$ is the confidence score of box $b_{i}$ for class *k*.

## 4. Results

### 4.1. Image Enhancement Effect Evaluation

**Figure 11:**
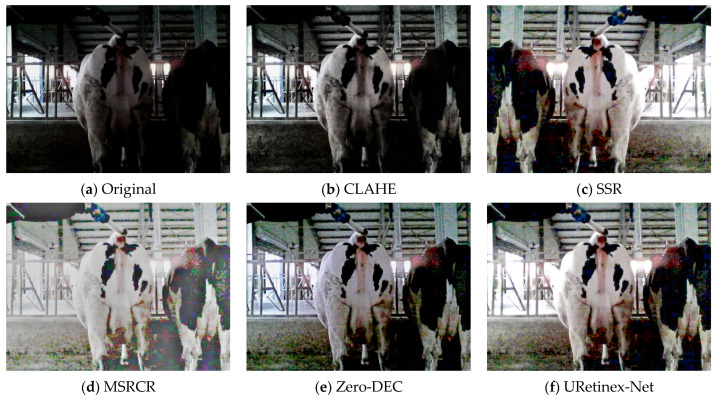
Comparison of different enhancement methods with original images in low-light conditions.

**Figure 12:**
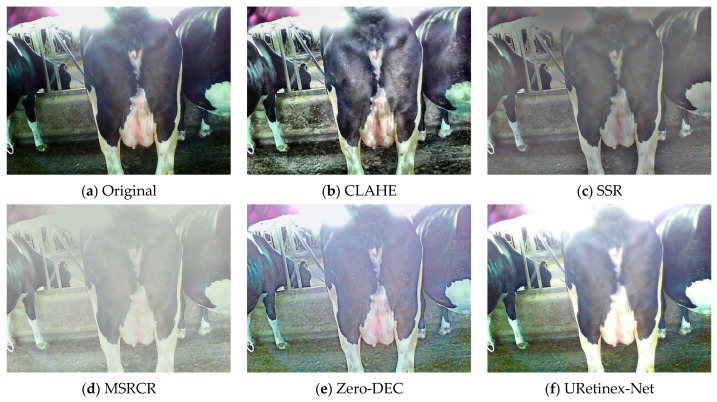
Comparison of different enhancement methods with the original images in overexposed conditions.

**Figure 13:**
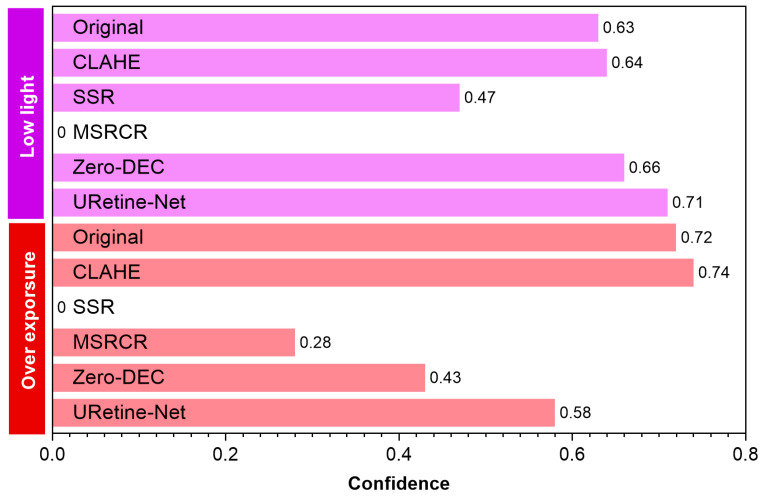
Comparison of detection performance between original images and enhanced images using the YOLO-World model.

This study employed manual observation to subjectively evaluate the enhancement effect (Figure 11 and Figure 12). Concurrently, YOLO-World was used to detect the images, with confidence as the objective evaluation index, to compare the detection performance of the original images and those processed by different enhancement methods (Figure 13).

For low-light images, images of cows exhibit significant reflectance differences because of their black-and-white pattern. White areas have higher brightness, while black areas have lower brightness and lose details. The CLAHE method has limited effectiveness in enhancing image brightness, and improvements in local contrast and brightness are not prominent. Even though SSR and MSRCR significantly enhance brightness, they result in severe loss of details in the processed images. Therefore, the confidence levels of these two methods are significantly reduced and, in some cases, no results are detected. Zero-DEC and URetinex-Net both effectively enhance image brightness. However, some differences exist between the two methods. Compared to the black-box method (Zero-DEC), URetinex-Net, which is based on physical modelling and decomposition, can better preserve structural and boundary information, making it more suitable for the fine-grained image enhancement task of cows with special skin colours. The confidence of the enhanced image is improved by 0.08 compared to the original image, which is 0.05 higher than Zero-DEC.

In overexposed scenes, SSR, MSRCR, and Zero-DEC methods can suppress brightness to some extent, but the enhanced images suffer from reduced contrast and colour distortion. Although the images processed by the URetinex-Net method exhibit minimal detail loss, they exhibit problems related to overexposure. This is because URetinex-Net lacks a brightness suppression constraint mechanism for overexposed regions. In overexposed scenes, the fur of cows is prone to uneven brightness reflection, which results in obvious brightness discontinuities. The key to solving this problem lies in enhancing local details while maintaining overall brightness coordination. CLAHE achieves fine-grained stretching within the brightness range via local enhancement and overall smoothing, alleviating brightness unevenness between regions. The confidence of the enhanced image is improved by 0.02 compared to the original image.

### 4.2. Performance Experiments of Different Models

**Figure 14:**
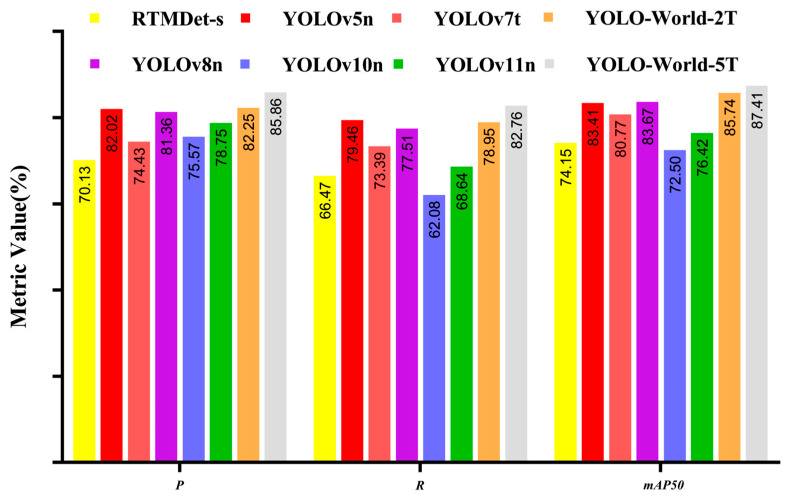
Comparison of detection performance results for different models.

RTMDet-s and several mainstream YOLO series models based on the MMCV framework were selected for detection performance comparison. Among them, YOLO-World-2T → 2C denotes a two-text, two-class object detection model, while YOLO-World-5T → 2C denotes a five-text, two-class object detection model. The LU and AA are the target categories for detection in the final model. Cow hind legs, RU, and hind sides act as intermediate auxiliary categories to establish spatial and semantic connectivity between the two ROIs. The training results of each model are presented in Figure 14.

**Figure 15:**
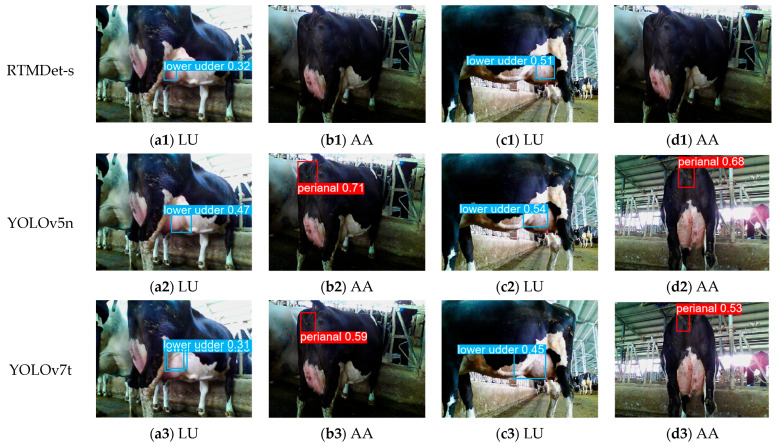

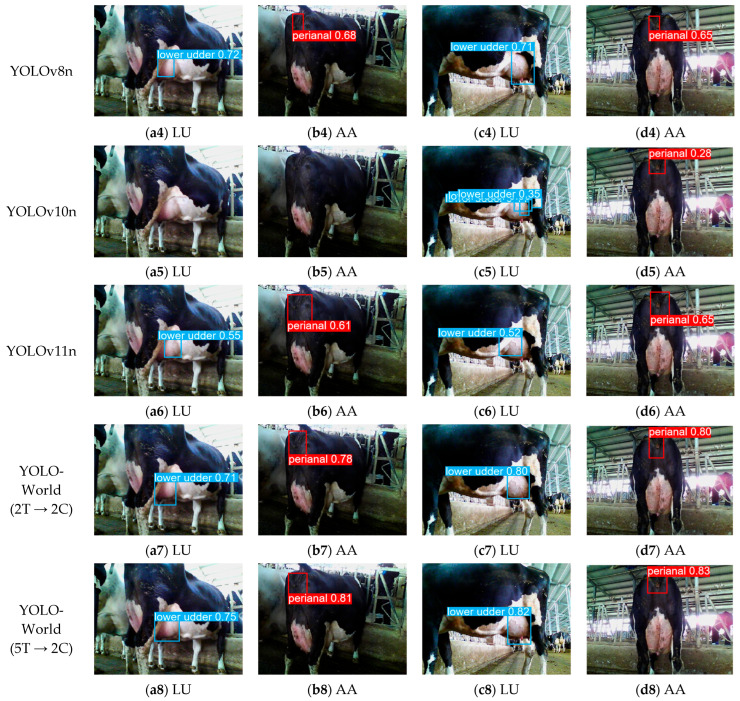
Comparison of confidence levels of images detected by different models.

Overall, RTMDet-s performs poorly across all metrics, with three evaluation metrics being significantly lower than the average of the YOLO series models. Among the YOLO series models, only YOLOv5, YOLOv8, and YOLO-World have P values greater than 80%. Although YOLOv5 and YOLOv8 models exhibit high detection accuracy, their detection results have significantly lower confidence levels compared to the YOLO-World model (Figure 15). Additionally, models trained on five-class text input outperform those trained on two-class text input across all metrics, including *P*, *R*, *mAP50*, and confidence, indicating that the richness of text modalities positively influences the detection performance of the model.

### 4.3. Effectiveness Evaluation of Multi-Text Input in Spatial Structure Relationship Modelling

The performance differences of the YOLO-World model under different numbers of text inputs were experimentally analysed. The number of text inputs is denoted as T and set to 2T, 3T, 4T, and 5T, representing the number of input texts. Concurrently, 2C indicates that only the detection results of the two categories LU and AA are counted in the inference stage (Table 5).

When using three text inputs, the addition of RU establishes an initial spatial connection between LU and AA, effectively creating a simplified connection path and enhancing the spatial perception capabilities of the model. This improves the *P* value; however, when ‘hind legs’ and ‘hind quarters’ were added, the limited semantic information resulted in ambiguous spatial positioning, failing to incorporate the LU and AA into the spatial relationship, resulting in no performance improvement.

When four text inputs are used, the spatial connections between different ROIs are more closely integrated, resulting in varying degrees of performance improvement across all models. The combination of the hindquarters and hind legs yields the best results. For five text inputs, the posterior region can integrate the first four ROIs into a unified structure, enabling the model to learn local spatial relationships from the overall structure. The final five-text model outperformed the two-text model with a 3.61% improvement in *P,* 3.81% improvement in *R*, and 1.67% improvement in *mAP50*. Two unrelated orthogonal vectors were transformed into related vectors by establishing semantic associations between the LU and AA using text labels, enhancing the accuracy of recognition in regions with blurred boundaries.

### 4.4. Comparison and Analysis of Visual Effects of Improved Modules Based on EigenCAM

**Figure 16:**
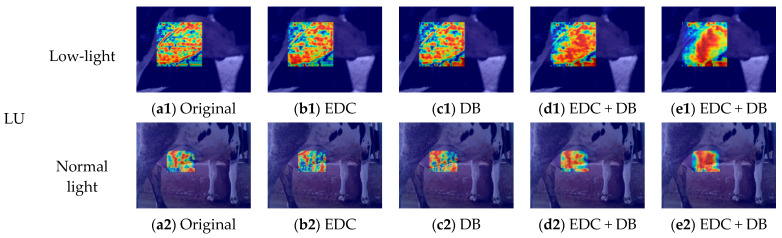

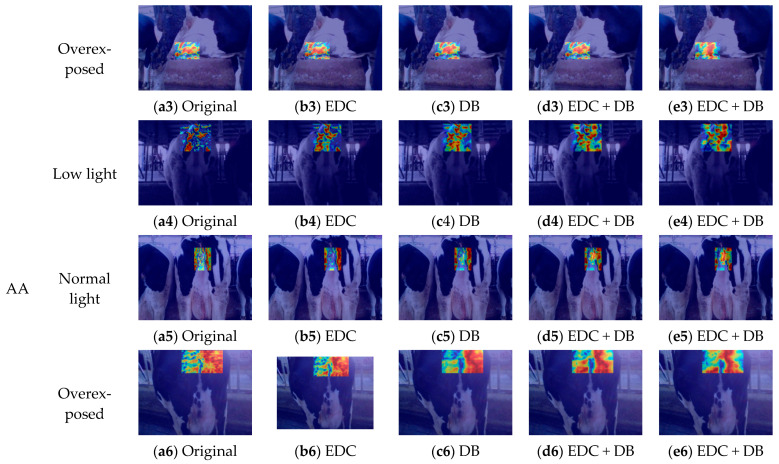
Ablation experiment results based on EigenCAM heat maps.

**Figure 17:**
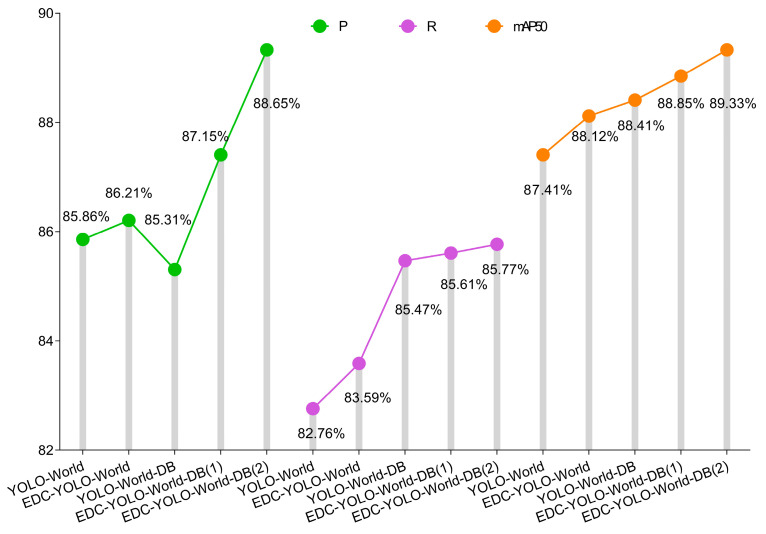
Ablation experiment results based on *P*, *R*, and *mAP50*.

Ablation experiments were conducted based on EigenCAM to validate the effectiveness of the proposed improvement method [44]. In the EigenCAM heatmaps, warmer colours (red and yellow) indicate regions with stronger model responses, whereas cooler colours (blue) indicate weaker responses. First, the original model was added to the ECD and BD modules separately to evaluate the independent contributions of each module. The two modules were then added to the model simultaneously to evaluate the improvement effect of the two modules working together. Finally, the effectiveness of the improved training strategy was compared, where EDC-YOLO-World (1) represents the original training strategy and EDC-YOLO-World (2) represents the improved training strategy (Figure 16 and Figure 17).

In experiments where the EDC and DB modules contributed independently, *R* and *mAP50* exhibit slight improvements compared to the original model, and the heat distribution in the heat map is more concentrated. However, following the introduction of the DB module, the *P* value of the model decreased slightly by 0.55%, indicating that weak association between text and image information results in the cross-scale fusion of image and text features from different levels; although helpful in identifying more true targets, it can also introduce a certain amount of false positives. This is because when the association between feature maps of different scales and text is insufficient, the model loses some of its ability to distinguish feature maps, resulting in the identification of interfering targets. Therefore, the heat distribution of DB in the heat map is not as concentrated as that of EDC. When the improved EDC and DB modules are added to the model simultaneously, *P*, *R*, and *mAP50* are all improved, the heat distribution is more focused on the ROI, and the brightness is significantly improved. This indicates that the fine segmentation ability of text is significantly improved under the synergistic action of the two modules, with good adaptability to feature maps of different scales.

Upon adopting an improved training strategy, the increase in the P value indicates that the TAM improved the recognition quality of positive samples, enhanced the alignment effect between tasks, and thereby enhanced the joint perception ability of target location and semantic information. For udder images with different angles and shapes, the heat feedback and ROI shapes show a high degree of overlap, and the heat distribution is closer to the actual contour of the ROI. The heat map distribution around the anus is closer to the centre-symmetric feature, which indicates the effectiveness of the Gaussian soft-constrained centre sampling strategy in guiding spatial distribution. Therefore, the matching quality of positive samples is also improved, which is reflected in the increase in the *R* value. The increase in *mAP50* indicates that the model can more accurately locate central structures in the AA and demonstrate stronger scale adaptability in the udder region, further optimising the overlap quality between prediction boxes and ground truth boxes. The improved model achieves a *P* value of 88.65%, an *R* value of 85.77%, and *mAP50* of 89.33%, representing improvements of 2.79%, 3.01%, and 1.92%, respectively, compared to the previous version.

### 4.5. Temperature Extraction and Validation

**Figure 18:**
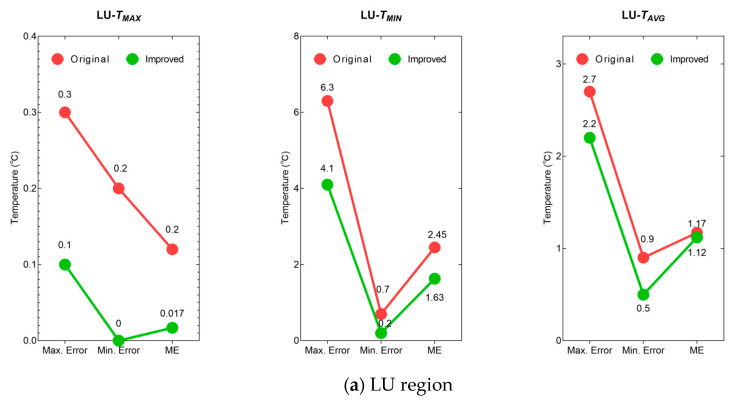

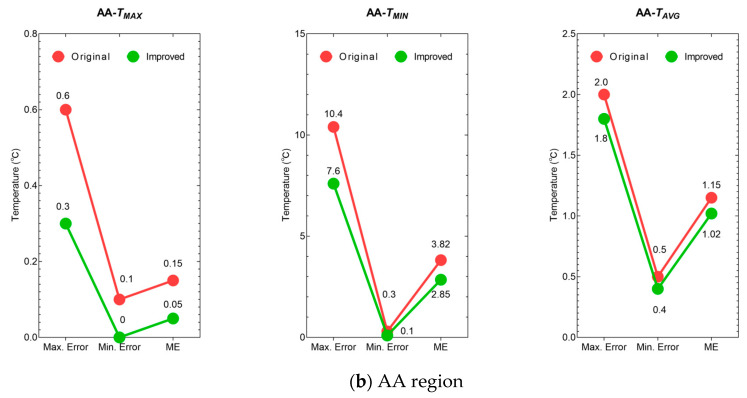
Results of three error evaluation metrics for LU and AA extraction temperatures.

This study used the EDC-YOLO-World-DB model with five text inputs to detect the AA and LU regions of dairy cows, and then performed temperature extraction on the ROIs of 36 cows to extract $T_{Max}$, $T_{Min}$, and $T_{avg}$ within the detection box. YOLO-World was used as the comparative experiment model (Figure 18). The evaluation metrics were set as the maximum error (Max. Error) between the extracted temperature of 36 cows and the actual temperature; the minimum error (Min. Error); and the mean error (ME).

Compared to the YOLO-World, the EDC-YOLO-World-DB model exhibits reduced errors across all temperature measurements. For the three indicators for LU: $T_{Max}$ decreased by 0.2 °C, 0.2 °C, and 0.183 °C, respectively; $T_{Min}$ decreased by 2.2 °C, 0.5 °C, and 0.82 °C, respectively; and $T_{avg}$ decreased by 0.5 °C, 0.4 °C, and 0.05 °C, respectively. AA: $T_{Max}$ decreased by 0.3 °C, 0.1 °C, and 0.1 °C respectively; $T_{Min}$ decreased by 2.8 °C, 0.2 °C, and 0.97 °C, respectively; $T_{avg}$ decreased by 0.2 °C, 0.1 °C, and 0.13 °C, respectively. In terms of relative reduction from ME, LU’s $T_{Max}$, $T_{Min}$, and $T_{avg}$ decreased by 66.6%, 33.5%, and 4.27%, respectively, while AA’s $T_{Max}$, $T_{Min}$, and $T_{avg}$ decreased by 66.6%, 25.4%, and 11.3%, respectively.

This study randomly selected six dairy cows and compared surface temperature values obtained via IRT under three lighting conditions: low light, normal light, and overexposed. These values are summarised in Table 6 to validate the practical applicability of the proposed system under varying light conditions. Temperature readings were recorded as the $T_{Max}$ within the ROI.

As shown in Table 6, all inaccurate detections only result in a slight underestimation of the true temperature, and no overestimation is observed. Across all three lighting conditions, the differences between actual and predicted temperatures for both LU and AA remain very small, and the predicted values closely follow the variation of the actual measurements. In particular, the relative temperature gradient between RT, LU, and AA is well preserved, indicating that the model can reliably capture physiologically meaningful changes in body temperature even under low-light and overexposed conditions. These results suggest that the proposed system provides stable and conservative temperature estimates under non-ideal illumination and, therefore, has promising potential for deployment in real farm environments for routine health monitoring and early screening.

From a physiological perspective, the observed gradient in which rectal temperature is highest, followed by the perianal surface (AA) and then the lower udder (LU), is consistent with the expected direction of heat transfer from the body core towards more peripheral tissues. The perianal region overlies the rectum and pelvic cavity and is richly vascularised, so increases in core temperature due to heat load or inflammatory processes are rapidly transmitted to this area via enhanced blood flow. By contrast, the lower udder has a larger exposed surface area and functions as an efficient site of convective and radiative heat loss, which tends to keep its surface temperature slightly lower than that of the AA region despite its proximity to the body core. The fact that the proposed model preserves these gradients and tracks subtle changes in AA and LU temperature suggests that its predictions are not only statistically robust but also anchored in physiologically meaningful thermoregulatory responses.

## 5. Discussion

The dual-path image enhancement, text-guided spatial structure modelling, EDC, DB, TAM, Gaussian soft-constrained centroid sampling, and hybrid *IoU* loss improvements proposed in this study effectively mitigate three issues prevalent in dairy barn environments: firstly, loss of texture and boundary information due to extreme lighting; secondly, colour interference and misclassification arising from the chaotic interplay of black-and-white patterns and backgrounds; thirdly, positioning inaccuracies and regression jitter caused by ROI structural deformation. The final model significantly enhances detection and localisation capabilities for the two critical ROIs—LU and AA—across diverse lighting conditions, specifically the following:

1. URetinex-Net and CLAHE respectively address detail loss in low-light images and brightness inconsistencies in overexposed images through image enhancement. Combined with subsequent cross-layer fusion via a bidirectional pyramid, this enhances overall ROI texture discernibility and boundary clarity while improving robustness to lighting variations.
2. Text-guided spatial structural relationships substantially mitigated colour interference and enhanced cross-scale consistency. Visualisation results demonstrate that text guidance concentrates heatmaps on structurally more plausible regions, while boundary alignment strategies better approximate actual anatomical contours. This indicates that weakly supervised semantic–geometric coupling effectively reduces errors stemming from visual discrepancies between colour and texture.
3. EDC and DB synergistically enhance the separability and reusability of cross-modal and cross-layer features. Ablation experiments demonstrate that introducing either EDC or DB alone yields modest improvements in *R* and *mAP50*. However, DB slightly reduces *P* values under weak correlations, indicating that cross-layer multimodal fusion requires robust semantic alignment capabilities. When both techniques are concurrently applied to refine the model, *P*, *R*, and *mAP50* improve synchronously, with heatmap distributions concentrating more sharply on the region of interest (ROI). This demonstrates the excellent enhancement effect of coupling dynamic semantic modulation with learning-based cross-layer fusion for fine-grained structural recognition.
4. The improved training strategy significantly enhances positive sample quality and localisation stability. This optimisation enables the final model to achieve *P* = 88.65%, *R* = 85.77%, and *mAP50* = 89.33%, representing improvements of 2.79%, 3.01%, and 1.92%, respectively, over the unmodified version.
5. With the proposed method, the errors in extracting $T_{Max}$, $T_{Min}$, and $T_{avg}$ were significantly reduced. Specifically, for LU, the ME of $T_{Max}$, $T_{Min}$, and $T_{avg}$ decreased by 66.6%, 33.5%, and 4.27%, respectively, compared to the original values; for AA, the ME of $T_{Max}$, $T_{Min}$, and $T_{avg}$ decreased by 66.6%, 25.4%, and 11.3%, respectively.
6. Beyond statistical performance, the temperature extraction results are also physiologically consistent. Under increased heat load, central thermoregulatory control adjusts peripheral blood flow so that changes in core temperature are rapidly reflected at the perianal surface, which lies close to the rectum and pelvic cavity, while the more peripheral and strongly heat-dissipating lower udder tends to remain slightly cooler. The preserved RT–AA–LU gradient and the close agreement between model-derived AA and LU temperatures and rectal temperature, therefore, indicate that the network is capturing meaningful thermoregulatory signals rather than merely fitting image-level patterns.

## 6. Limitations and Future Research

1. Real-time Performance: The image enhancement processing procedure impacts the real-time detection capability of the model. Future research will explore lightweight end-to-end micro-enhancement strategies and incorporate acceleration techniques such as NPU/FP16/INT8.
2. Occlusion: Tail wagging and dirt coverage may trigger false positives or negatives. Future research will incorporate temporal and short-term trajectory correlation to mitigate single-frame image uncertainty.
3. Textual Prior Quality: The detail and accuracy of textual descriptions influence EDC’s weight distribution effectiveness. Subsequent research will establish more precise and verifiable anatomical corpora, alongside automated text enhancement and alignment mechanisms.
4. Temperature error: Irrelevant pixels may lead to elevated errors in $T_{Min}$ and $T_{avg}$. Future research will focus on refining the ROI segmentation of dairy cows to minimise the potential for interference from extraneous pixels.
5. Fur colour pattern: In Holstein cows, the udder is predominantly white but may contain small black patches and is connected to surrounding irregular black–white fur patterns. Even these occasional dark areas can noticeably degrade model performance, as the network can only learn from shape cues and two fur colours. This study mainly addresses the effect of small black patches and black–white junctions around the udder; future research will further refine the model for cows with predominantly white udders.

## 7. Conclusions

In this study, the LU and AA were identified as the most reliable ROIs for tracking changes in RT under variable lighting conditions. Across all experiments, the EDC-YOLO-World-DB model demonstrated the most favourable performance, achieving a *P* value of 88.65%, an *R* value of 85.77%, and an *mAP50* of 89.33%., Compared with the original YOLO-World model, the errors in LU and AA surface temperature extraction were markedly reduced, with mean errors of key temperature indices decreasing by approximately 25–65% in both regions. These results indicate that the proposed system can be deployed above passageways or milking areas to continuously track LU and AA temperatures in commercial herds, enabling automatic identification of cows with abnormal RT for early health screening management without additional handling.

## Figures and Tables

**Table 1:** FLIR-E6 IR Thermal imager specifications and configuration.

Parameter	Value
RGB pixels	640 × 480
Infrared resolution	320 × 240
Emission	0.98
Field of view	45° × 34°
Spatial resolution	3.7 mrad
Thermal sensitivity NETD ^1^	50 mK
Temperature measurement range	−20–250 °C

^1^ NETD: noise equivalent temperature difference.

**Table 2:** Dataset division.

Dataset Structure	Illumination Conditions		
	Low Light	Normal Light	Overexposed
Training set	733	955	624
Validation set	245	318	208
Test set	245	318	208

**Table 3:** Example rectal and surface temperatures of ten lactating dairy cows.

Cow ID	RT (°C)	LU (°C)	AA (°C)
Cow 1	38.9	36.5	37.6
Cow 2	38.8	36.7	37.4
Cow 3	39.0	37.2	38.1
Cow 4	38.2	36.4	37.1
Cow 5	38.6	35.2	37.2
Cow 6	38.6	35.7	37.3
Cow 7	38.8	36.5	37.2
Cow 8	38.4	36.2	37.4
Cow 9	38.4	36.4	36.9
Cow 10	39.7	37.5	38.5

**Table 4:** Description of the five categories of texts.

Text	Description
LU	Next to the hind leg of the cow, where the hind leg connects to the RU, the RU is not visible when the LU is visible
AA	Located at the top of the RU, above the hindquarters, adjacent to the stifle
RU	From the rear of the cow, it can be observed located below the AA region, adjacent to the hind legs, and connected to the LU at the lowest point
Hind legs	Next to the LU, RU, and AA
Hind quarters	Including the AA, upper side of the tail, and entire hindquarters

**Table 5:** Text combination for modelling spatial structural relationships.

Text	ROI					Results		
	LU	AA	RU	Hind Legs	Hind Quarters	*P*	*R*	*mAP50*
2T → 2C	√ ^1^	√	× ^2^	×	×	82.25	78.95	85.74
3T → 2C	√	√	√	×	×	83.63	78.64	83.09
	√	√	×	√	×	82.25	78.95	85.74
	√	√	×	×	√	82.25	78.95	85.74
4T → 2C	√	√	√	√	×	84.77	81.84	85.61
	√	√	√	×	√	83.13	80.32	84.97
	√	√	×	√	√	83.77	81.84	85.61
5T → 2C	√	√	√	√	√	85.86	82.76	87.41

^1^ √ indicates that the corresponding ROI text category is included in the model input. ^2^ × indicates that it is not included.

**Table 6:** Example rectal and surface temperatures of ten cows under different lighting conditions.

Condition	RT (°C)	Actual Temperature (°C)		Predicted Temperature (°C)	
		LU	AA	LU	AA
Low light	38.6	35.2	37.2	35.1	37.2
	38.6	35.7	37.3	35.7	37.2
Normal light	38.9	36.5	37.6	36.4	37.5
	38.8	36.7	37.4	36.7	37.4
Overexposed	38.4	36.4	36.9	36.3	36.7
	39.7	37.5	38.5	37.4	38.2

## Data Availability

The data presented in this study are available on request from the corresponding authors.
